# A comparison of two methods for estimating measurement repeatability in morphometric studies

**DOI:** 10.1002/ece3.7032

**Published:** 2021-01-06

**Authors:** Zachariah Wylde, Russell Bonduriansky

**Affiliations:** ^1^ Evolution and Ecology Research Centre School of Biological, Earth and Environmental Sciences University of New South Wales Sydney NSW Australia

**Keywords:** allometry, measurement error, phenotype, precision, repeatability

## Abstract

1. Measurement repeatability is often reported in morphometric studies as an index of the contribution of measurement error to trait measurements. However, the common method of remeasuring a mounted specimen fails to capture some components of measurement error and could therefore yield inflated repeatability estimates. Remounting specimens between successive measurements is likely to provide more realistic estimates of repeatability, particularly for structures that are difficult to measure.
2. Using measurements of 22 somatic and genitalic traits of the neriid fly *Telostylinus angusticollis*, we compared repeatability estimates obtained via remeasurement of a specimen that is mounted once (single‐mounted method) versus remeasurement of a specimen that is remounted between measurements (remounted method). We also asked whether the difference in repeatability estimates obtained via the two methods depends on trait size, trait type (somatic vs. genitalic), sclerotization, or sex.
3. Repeatability estimates obtained via the remounted method were lower than estimates obtained via the single‐mounted method for each of the 22 traits, and the difference between estimates obtained via the two methods was generally greater for small structures (such as genitalic traits) than for large structures (such as legs and wings). However, the difference between estimates obtained via the two methods did not depend on trait type (genitalic or somatic), tissue type (soft or sclerotized) or sex.
4. Remounting specimens between successive measurements can provide more accurate estimates of measurement repeatability than remeasuring from a single mount, especially for small structures that are difficult to measure.

## Graphical abstract

Measurement repeatability is often reported in morphometric studies as an index of the contribution of measurement error to trait measurements. The common method of remeasuring a mounted specimen fails to capture some components of measurement error however, and could therefore yield inflated repeatability estimates. Using a data set of insect morphology, we show that remounting specimens between successive measurements can provide more accurate estimates of measurement repeatability than remeasuring from a single mount, especially for small structures that are difficult to measure.

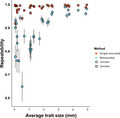

## 1 INTRODUCTION

Studies that focus on morphology or that utilize morphometrics in some way have a long tradition within the fields of ecology and evolution. The development of reliable and standardized methods for measuring morphology is an important but often overlooked challenge in evolutionary ecology (Kozlov, 2015). Yet, relatively little is known as to how much variation in the findings of morphological studies, particularly on small organisms such as insects, is the result of differences in measurement protocol.

A key source of variation in morphometric analysis is measurement repeatability. Repeatability is estimated from repeated measurements taken on several individuals and is typically calculated as the intraclass correlation coefficient—that is, the proportion of total variance that is attributable to individual identity (Lessells & Boag, 1987; Sokal & Rohlf, 1995; Stoffel et al., 2017). Repeatability is estimated for many different reasons (Wilson, 2018). In morphometric studies, repeatability is useful as a gauge of the contribution of measurement error to trait measurements, and therefore the statistical power of analyses involving those measurements (Bailey & Byrnes, 1990; Yezerinac et al., 1992). In principle, if Trait A has greater measurement error than Trait B, Trait A will have lower repeatability than Trait B, and analyses of variation in Trait A will have lower statistical power. While this is true for fixed morphological traits, for repeatability of behavioral or life‐history traits, the residual variance will include both measurement error and other processes such as reversible plasticity (i.e., temporal environmental effects) (Dingemanse & Dochtermann, 2014; Westneat et al., 2015). From a statistical standpoint, the calculation of repeatability has received considerable attention in the literature (Altaye et al., 2001; Ghosh & Das, 2003; Lessells & Boag, 1987; Nakagawa & Schielzeth, 2010; Shoukri & Donner, 2001; Stoffel et al., 2017).

Specimen handling and remeasurement methods can also affect repeatability estimates. For example, a study on the morphometrics of skeletal traits in passerine birds found that, as measuring technique improved through experience, measurement error declined (Yezerinac et al., 1992). The repeatability of the trait being measured can depend on both genetic and extrinsic factors (Wilson, 2018). The way in which one handles and measures a specimen is likely to be an important but somewhat overlooked extrinsic factor that also influences repeatability. Repeatability can be particularly tricky to estimate properly, especially for small samples with few repeated measures (Dingemanse & Dochtermann, 2013). Guidelines exist on the number of individuals and number of measurements per individual required to obtain precise estimates of repeatability (Wolak et al., 2012), but guidelines on appropriate specimen handling and remeasurement techniques are currently lacking.

To provide useful estimates of the contribution of measurement error to trait measurements, repeatability estimates must capture as many sources of measurement error as possible. In morphometric studies (particularly those involving small specimens, such as insect body parts), measurement error typically reflects how specimens are mounted, and how their dimensions are quantified. A common procedure is to remeasure a single mount (or an image of a single mount). This method has been used widely in studies by our lab and other groups (Bertin & Fairbairn, 2007; Blanckenhorn et al., 2004; Bonduriansky, 2007; Bonduriansky et al., 2015; Cayetano & Bonduriansky, 2015; Hosken et al., 2005). Many other morphometric studies do not specify how repeatability was estimated, or do not report repeatability at all. However, this method omits sources of measurement error associated with specimen mounting. For example, each time a specimen is mounted its orientation relative to the focal plane of the microscope or camera will be slightly different, resulting in different degrees of parallax error. Soft specimens may also be distorted slightly each time they are handled, and images of specimens mounted in fluid medium (such as glycerol or saline solution) may be shadowed or distorted in various ways by the fluid.

Remounting specimens between successive measurements is therefore likely to yield better estimates of measurement repeatability. However, remounting takes time and effort, and it is not clear how substantially estimates of repeatability obtained by remounting would differ from estimates obtained by remeasuring the same mount, or whether the difference between estimates obtained by these methods varies between traits that can be measured with relatively little error (such as large, flat and stiff morphological structures) and traits that are subject to greater measurement error (such as small or soft morphological structures). To address these questions, we compared repeatability estimates obtained via remeasuring a single mount (single‐mounted method) versus estimates obtained via remounting (remounted method) for 22 morphological traits of the neriid fly *Telostylinus angusticollis*. The traits included small and weakly sclerotized genitalic structures as well as relatively large, strongly sclerotized and flat structures such as legs and wings.

## 2 MATERIAL AND METHODS

### 2.1 Study system

We utilized a morphometric dataset on the neriid fly *Telostylinus angusticollis* that included 22 somatic and genitalic traits measured on individuals reared on nutrient‐rich and nutrient‐poor larval diets (Wylde & Bonduriansky, 2020). Eggs collected from stock flies were reared using a larval diet that is intermediate in nutrient concentration between the rich and poor diets, based on Sentinella et al. (2013). Randomly chosen adults were then paired to create 17 mating pairs. From each pair, 20 eggs were transferred to the poor larval diet and 20 eggs were transferred to the rich larval diet. Adults emerging from these larval diets were frozen for measurement ~24 hr after emergence (i.e., once their exoskeletons had sclerotized fully). Larval diet manipulation influences adult body size and shape in *T. angusticollis* (Bonduriansky, 2007), and therefore increased the range of variation in the sizes of morphological traits examined in this study.

**FIGURE 1:**
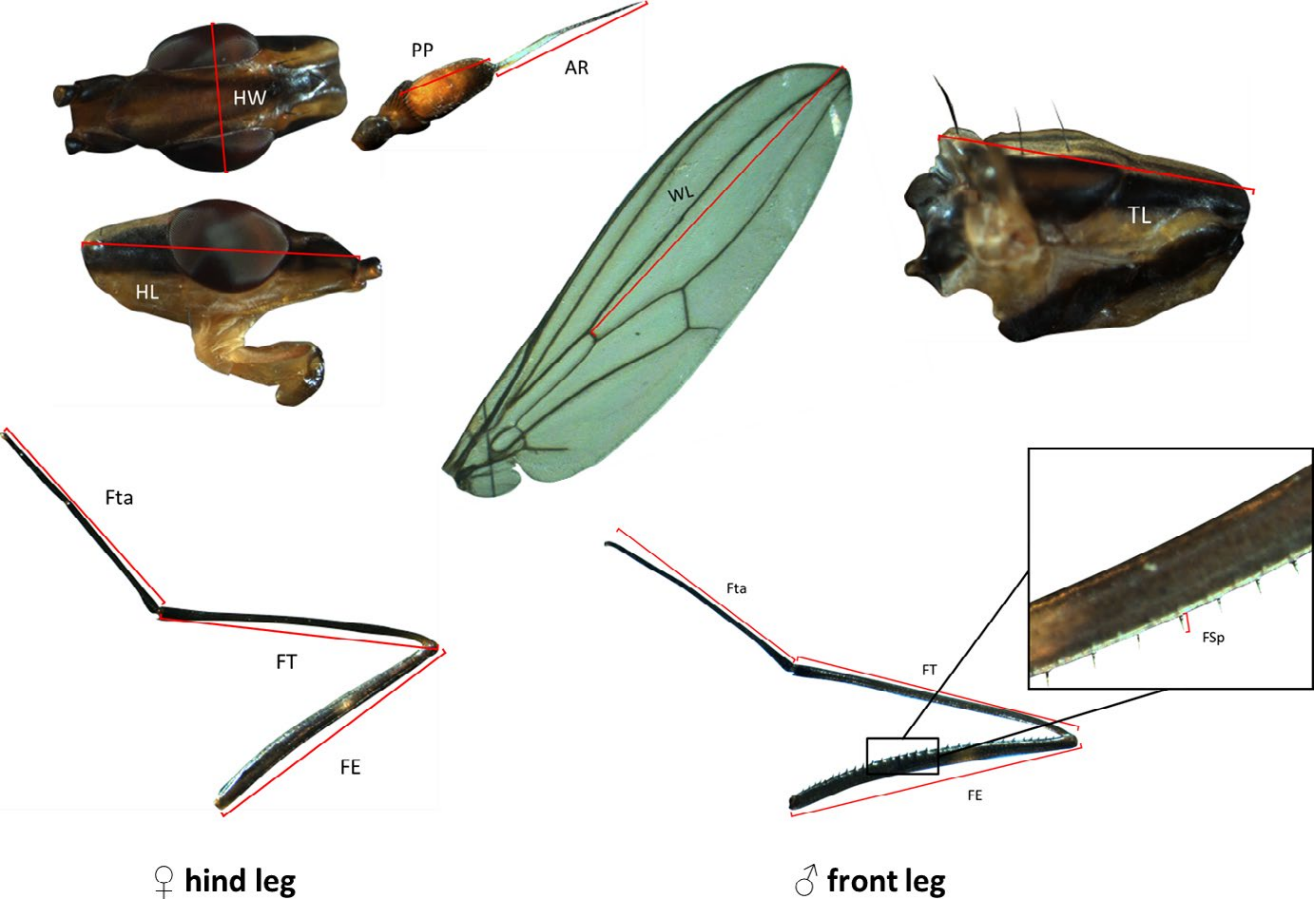
Larger sclerotized body parts of *Telostylinus angusticollis*. Shared traits between male and female include HW (head width), HL (head length), PP (postpedicel length), AR (arista length), WL (Wing length) and TL (thorax length). In females, the hind leg was measured because of its role in female–male interactions, whereas in males, the front leg was measured because of its involvement in male–male combat. Leg measurements comprised FE (femur length), FSp (femur spine length, males only), FT (tibia length), Fta (tarsus length). Trait images are not to scale

**FIGURE 2:**
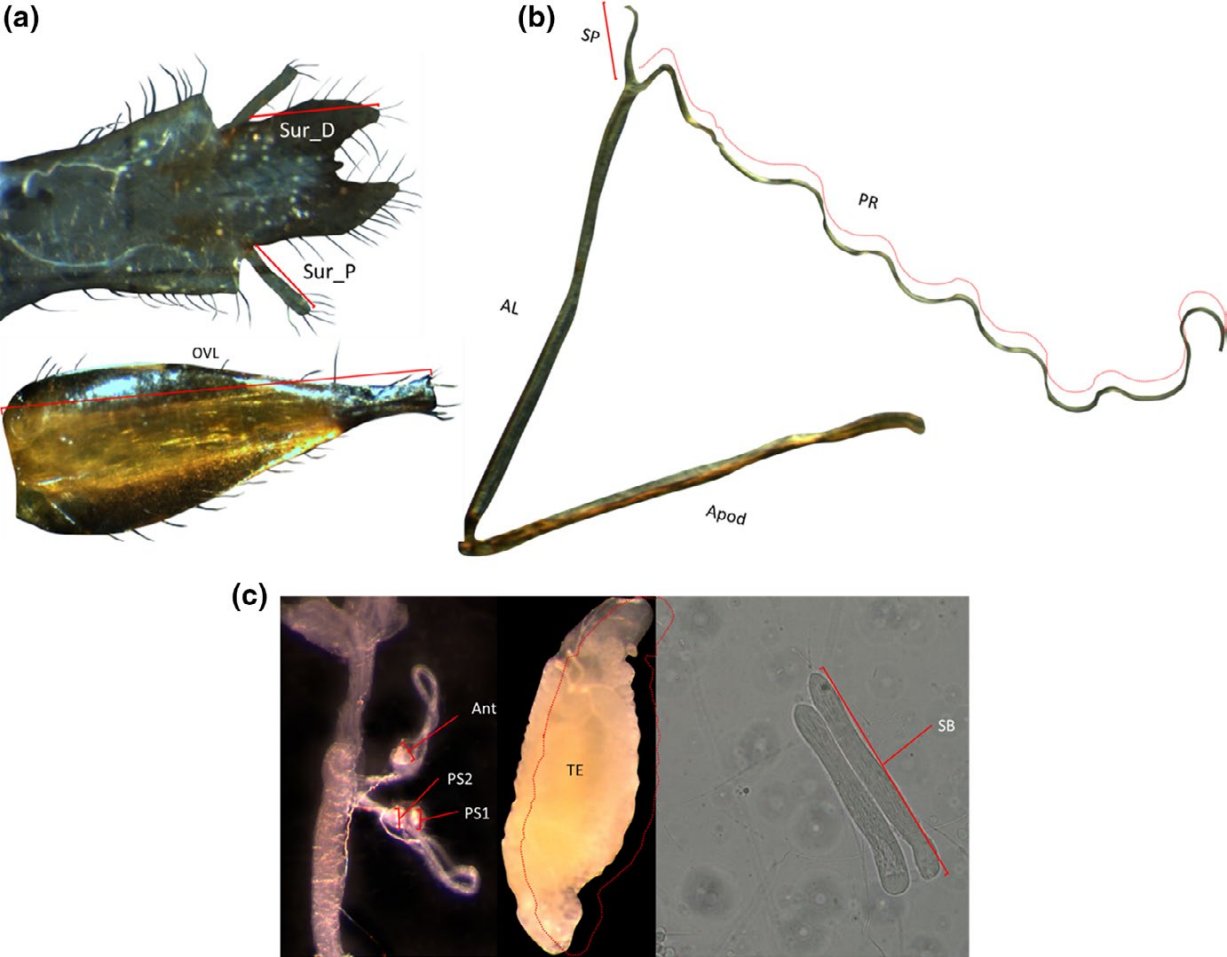
Genitalic and other smaller and unsclerotized traits of *Telostylinus angusticollis*. (a) External genitalic traits: male Sur_P (proximal surstyli length) and Sur_D (distal surstyli length); female oviscape (OVL). (b): Male genitalic apparatus: Apod (apodeme length), AL (Aedeagus length), SP (short anterior processus length), PR (processus length). (c): left, female genitalic apparatus: PS1, PS2 (posterior spermathecae 1 &amp; 2 width), Ant (anterior spermatheca width); male TE (testis, area mm^2^) and SB (sperm bundle length)

We measured six genitalic and 12 somatic traits on each of 93 males (*n* = 43 poor diet, *n* = 50 rich diet), and four genitalic and 11 somatic traits on each of 96 females (*n* = 49 poor diet, *n* = 47 rich diet). All trait measurements were lengths in mm except for testis area, which was measured in mm^2^ (see Figures 1 and 2 for definitions of trait measurements). Two methods were used to estimate repeatability for each trait. First, each specimen was mounted on the slide and imaged, and then remounted and reimaged; separate measurements were then made from the two images (“remounted” method). Second, each trait was measured twice from a single image (“single‐mounted” method). For the single‐mounted method, we chose which of the two images to remeasure based on a random sequence of numbers generated from a binomial distribution.

### 2.2 Sample preparation

For each individual, the head, wings, legs and antennae were separated from the thorax and the genitalia were dissected out. Body parts were laid flat onto 1–1.2 mm microscope slides (ISSCO®) with an in‐built micrometer for measurement calibration. To minimize parallax error, heads were positioned on slides covered with double‐sided tape. Genitalic structures were mounted in 7.2 pH Phosphate Buffered Saline (PBS) and covered with 22 mm coverslips. The external genitalia (epandrium), male surstyli (proximal and distal) and internal section of the genitalia (carefully removed as one unit that included the apodeme, aedeagus and processes) were separated from the epandrium and placed under a coverslip. All somatic (both sexes) and male genitalic traits were imaged using a Leica MZ 16A stereoscope fitted with a Leica MC170 HD camera. Before dissection of spermathecae, the female oviscape was imaged and its length measured. The female reproductive tract with spermathecae was then carefully removed, cleaned and mounted in PBS as described above. In order to better observe boundaries of the spermathecal structures, which are translucent and substantially smaller and softer than the other traits measured, we imaged all spermathecae using a Zeiss Axioskop 40 compound microscope fitted with a DinoEyepiece® camera at 200× magnification.

### 2.3 Statistical analysis

All analyses were carried out using R 3.6.2 (R Core Team, 2019). Repeatability was calculated for each trait as the variance among individual trait means (individual‐level variance V*_i_*) over the sum of individual‐level and residual variance *R* = V*_i_*/( V*_i_* + V*_R_*). We split the data by sex and method of measurement (single‐mounted or remounted) and fit separate linear mixed models using the packages “lmerTest” (Bates et al., 2015; Kuznetsova et al., 2017) and “lme4” (Bates et al., 2015) where trait size was the response variable, larval diet (rich or poor) was the fixed categorical predictor, and trait ID was the random effect. Subsequently, we used parametric bootstrapping (1,000 iterations, 500 permutations) to obtain uncertainty in estimated repeatability values using the package “rptR” (Stoffel et al., 2017). We first compared the bootstrapped distributions obtained from the two measurement methods using a pairwise Wilcoxon test using the package “rstatix” (Kassambara, 2020) and “coin” (Hothorn et al., 2006) to calculate effect size (*r*).

We then fit a linear mixed model to the mean repeatability estimates, with method (single‐mounted vs. remounted) as a fixed categorical predictor, mean trait size, and trait type (sclerotized vs. soft) as fixed covariates, and all two‐way interactions of these predictors with method. Trait ID was included in the model as a random effect. Conditional effect sizes reflect the variance explained by both the fixed and random effects while marginal effect sizes reflect the fixed effects only (Nakagawa & Schielzeth, 2013). These metrics allow us to quantify the magnitude of the influence that each factor has on the dependent variable (mean repeatability). We therefore ran separate models for each fixed effect and interaction to calculate marginal and conditional effect sizes using the technique of Nakagawa and Schielzeth (2013) (outlined above).We calculated effect sizes using the “piecewiseSEM” package (Lefcheck, 2016). We found some evidence of deviations from residual normality (see Figure S1). We therefore also tested the effects of categorical predictors (trait type, tissue type, sex) and their interactions with method using nonparametric ANOVA based on aligned rank transformation, calculated with the package “ARTool” (Kay & Wobbrock, 2020) (see supplementary information). We did not test trait size or the method x trait size interaction using this approach because nonparametric ANOVA cannot be used with continuous predictors.

Our analysis assumes that any difference between repeatability estimates obtained via single‐mounting versus remounting methods reflects a difference in residual variance of repeated measurements. To test this assumption, we directly modeled changes in residual variance (sigma) as a function of measurement method, using the packages “rstan” (Stan Development Team, 2020) within the Bayesian framework “brms” (Bürkner, 2018). We lacked sufficient power to test the full model with all interactions using “brms.” We therefore used brms to model only the fixed effect of method with Trait ID as the random effect. Posterior distributions of “brms” model parameters were generated from 3,000 iterations (following 1,000 burn‐in iterations) spread across two chains (see the Appendix S1).

## 3 RESULTS

**FIGURE 3:**
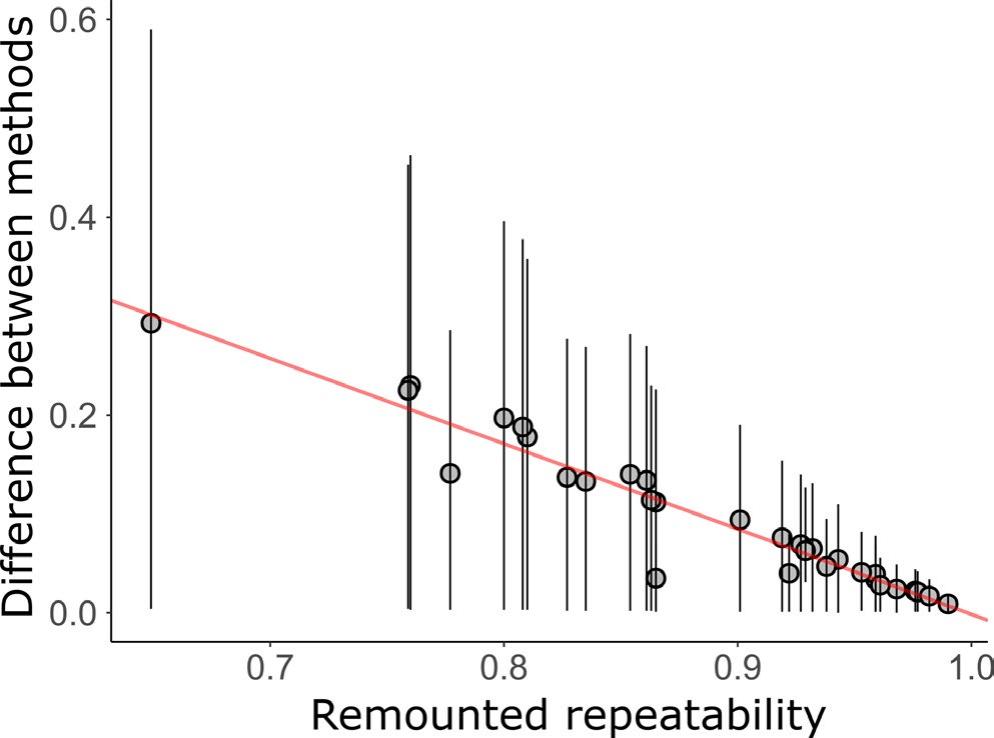
The difference between repeatability estimates obtained via single‐mounted and remounted methods increases as remounted repeatability decreases. Bars represent 95% confidence intervals obtained from bootstrapped repeatability estimates

Repeatability estimates obtained via remounting were lower than estimates obtained via single‐mounting for each of the 22 traits (Table 1) (pairwise Wilcoxon signed‐rank test, *v* = 1, *p* < .001, *r* = .866). The difference between repeatability estimates obtained via single‐mounted and remounted methods (Δ*R*) increased as remounted repeatability decreased (*r* = −0.98, *t* = −28.1, *p* < .001; Figure 3). This illustrates that as measurement accuracy decreases in remounted estimates, repeatability estimates become more inflated when using the traditional, single‐mounted method.

**TABLE 1:** Estimates of trait repeatability based on remounting and single‐mounting methods for 22 morphometric traits of *Telostylinus angusticollis*

Trait abbreviation	Mean trait size (mm)	Repeatability (single‐mounted) [95% CI]	Repeatability (remounted) [95% CI]	Δ*R* [95% CI]
**Female somatic traits**				
PP	0.519	0.941 [0.914, 0.960]	0.649 [0.501, 0.759]	0.293 [0.289, 0.297]
AR	1.05	0.987 [0.980, 0.992]	0.810 [0.728, 0.872]	0.178 [0.175, 0.180]
HW	1.309	0.993 [0.990, 0.996]	0.861 [0.800, 0.906]	0.134 [0.132, 0.136]
HL	1.705	0.993 [0.989, 0.995]	0.854 [0.788, 0.903]	0.140 [0.138, 0.142]
TL	2.34	0.994 [0.991, 0.996]	0.927 [0.889, 0.953]	0.069 [0.068, 0.071]
Fta	2.589	0.992 [0.988, 0.995]	0.968 [0.950, 0.980]	0.024 [0.024, 0.025]
WL	3.46	0.999 [0.999, 0.999]	0.990 [0.984, 0.994]	0.009 [0.009, 0.010]
FT	3.743	0.992 [0.988, 0.995]	0.959 [0.939, 0.973]	0.034 [0.033, 0.034]
FE	4.354	0.997 [0.996, 0.998]	0.976 [0.964, 0.985]	0.022 [0.021, 0.022]
**Female genitalic traits**				
Ant	0.063	0.995 [0.992, 0.997]	0.901 [0.847, 0.939]	0.094 [0.093, 0.096]
PS1	0.073	0.995 [0.993, 0.997]	0.932 [0.892, 0.957]	0.065 [0.064, 0.066]
PS2	0.075	0.994 [0.991, 0.996]	0.919 [0.876, 0.949]	0.076 [0.075, 0.078]
OVL	1.946	0.991 [0.987, 0.994]	0.929 [0.893, 0.952]	0.063 [0.032, 0.064]
**Male somatic traits**				
FSp	0.051	0.919 [0.881, 0.946]	0.777 [0.672, 0.859]	0.141 [0.138, 0.145]
SB	0.161	0.994 [0.990, 0.996]	0.760 [0.655, 0.847]	0.230 [0.227, 0.233]
TE	0.378	0.996 [0.995, 0.998]	0.943 [0.906, 0.965]	0.054 [0.054, 0.056]
PP	0.659	0.975 [0.963, 0.983]	0.865 [0.795, 0.913]	0.112 [0.110, 0.114]
AR	1.077	0.994 [0.991, 0.996]	0.800 [0.690, 0.871]	0.197 [0.194, 0.199]
HW	1.316	0.993 [0.990, 0.996]	0.865 [0.794, 0.912]	0.035 [0.034, 0.036]
HL	2.24	0.998 [0.997, 0.999]	0.922 [0.881, 0.950]	0.040 [0.039, 0.040]
TL	2.545	0.997 [0.996, 0.998]	0.959 [0.937, 0.974]	0.039 [0.038, 0.039]
Fta	3.583	0.993 [0.989, 0.996]	0.953 [0.925, 0.972]	0.041 [0.039, 0.041]
WL	3.671	0.998 [0.997, 0.999]	0.977 [0.964, 0.986]	0.021 [0.020, 0.021]
FT	4.877	0.988 [0.982, 0.992]	0.961 [0.938, 0.975]	0.028 [0.027, 0.028]
FE	4.946	0.999 [0.998, 0.999]	0.982 [0.972, 0.989]	0.017 [0.016, 0.017]
**Male genitalic traits**				
SP	0.17	0.977 [0.964, 0.985]	0.863 [0.794, 0.912]	0.114 [0.112, 0.116]
Sur_P	0.177	0.967 [0.949, 0.978]	0.835 [0.750, 0.894]	0.133 [0.131, 0.136]
Sur_D	0.295	0.980 [0.970, 0.987]	0.759 [0.642, 0.843]	0.225 [0.221, 0.228]
Apod	1.091	0.964 [0.946, 0.977]	0.827 [0.736, 0.889]	0.137 [0.135, 0.140]
AL	1.136	0.996 [0.994, 0.998]	0.808 [0.712, 0.879]	0.188 [0.185, 0.190]
PR	1.864	0.984 [0.976, 0.990]	0.938 [0.902, 0.962]	0.047 [0.046, 0.048]

Note

Traits are ordered by sex, type (genitalic vs. somatic), and increasing mean size (mm). Δ*R* represents the difference between repeatability estimates obtained via single‐mounting versus remounting methods.

**FIGURE 4:**
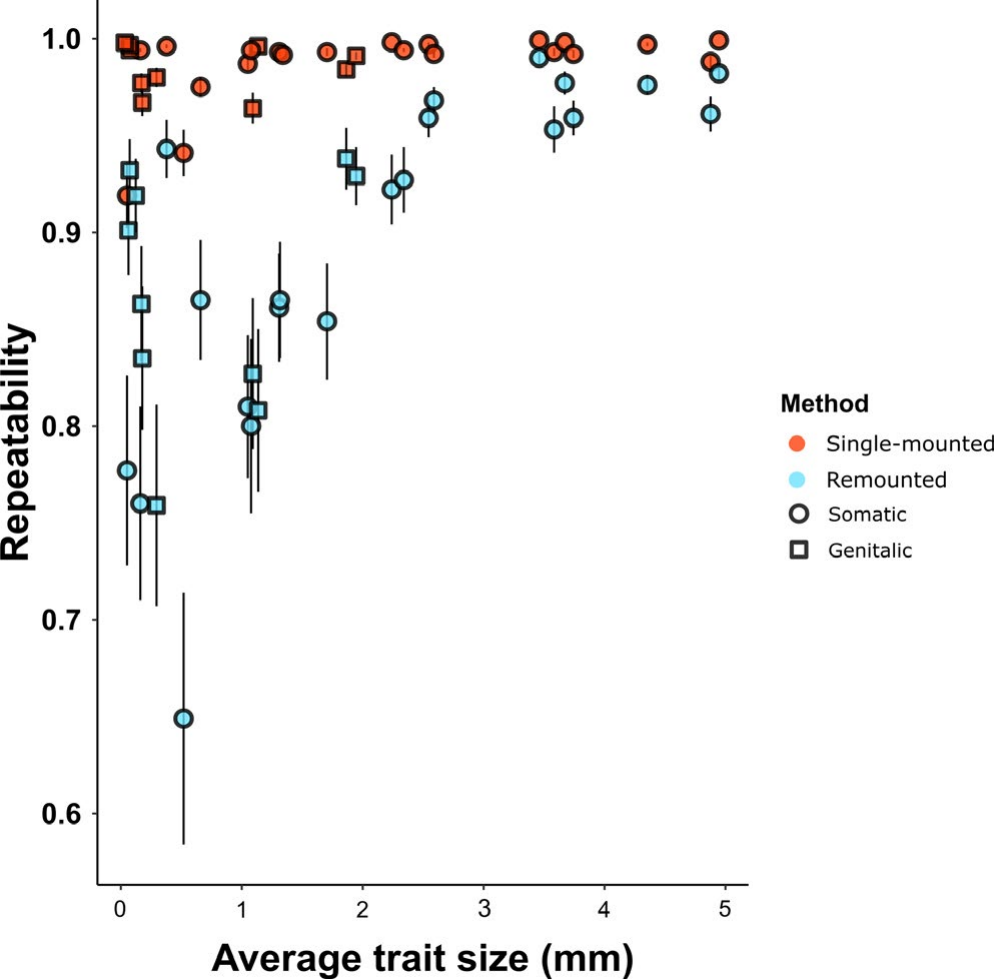
Estimates of repeatability obtained by remounted and single mount methods, arranged in order of increasing mean trait size (smallest to largest, left to right). Bars represent standard error of the mean calculated from bootstrapped estimates of repeatability

Small or unsclerotized structures might be more difficult to measure accurately. Therefore, we also asked whether several trait characteristics might influence repeatability estimates obtained using the two methods. Because the repeatability estimates deviate to some extent from assumptions of parametric testing, we base our interpretation on two approaches. First, we used a generalized linear mixed model to test effects on repeatability of method (single‐mounted vs. remounted), as well as sex, trait size, trait type (genitalic vs. somatic), tissue type (sclerotized vs. unsclerotized), and 2‐way interactions of these trait characteristics with method. In addition, we tested the categorical predictors (sex, trait type, tissue type) and their interactions with method using nonparametric ANOVA. We found a significant interaction between trait size and measurement method whereby Δ*R* was greater for smaller traits (Table 2). Thus, as traits increased in size and therefore decreased in average difficulty of consistent measurement, differences between the two methods decreased (Figure 4). For trait type, tissue type and sex, the impact of method on repeatability was less clear. For tissue type, the mixed model suggested that sclerotized tissues had higher repeatability estimates than soft tissues when single‐mounted but not when remounted, but this interaction was not supported by nonparametric ANOVA (Figure S2b). Neither the mixed model nor the nonparametric ANOVA provided support for interactions of measurement method with sex or trait type (Table 2, Table S1, Figure S2).

**TABLE 2:** Generalized linear mixed effects model of bootstrapped distributions of repeatability with marginal and conditional effect sizes

	Estimate	Std. Error	*df*	*t*	*p*	*R* ^2^_GLMM(_ *_m_*_)_	*R* ^2^_GLMM(_ *_c_*_)_
(Intercept)	0.799	0.019	51.963	41.650	<.001	—	—
Method	0.173	0.027	36.514	6.515	<.001	41.92%	60.24%
Mean trait size	0.051	0.006	41.046	9.019	<.001	15.86%	15.86%
Sex	−0.014	0.014	45.653	−1.003	.321	0.66%	1.01%
Tissue type (sclerotized vs. unsclerotized)	0.088	0.020	50.956	4.406	<.001	0.21%	3.36%
Trait type (genitalic vs. somatic)	−0.020	0.018	50.044	−1.139	.260	0.57%	2.85%
Method × Mean trait size	−0.044	0.008	36.514	−5.671	<.001	65.85%	73.05%
Method × Sex	0.010	0.020	36.514	0.514	.611	41.58%	59.12%
Method × Tissue type	−0.070	0.027	36.514	−2.552	.015	41.41%	60.25%
Method × Trait type	0.019	0.025	36.514	0.784	.438	41.78%	60.40%

Note

The intercept is compared against the remounted method.

Finally, as expected, ΔR reflected differences in residual variance of repeated measurements rather than differences in the variance of individual trait means (see Appendix S1).

## 4 DISCUSSION

We found that both sample‐handling method and the size of the trait influenced estimates of repeatability. For each of the 22 traits examined, we found that repeatability estimates obtained by remounting samples between successive measurements were smaller than repeatability estimates obtained by remeasuring a single mount. Moreover, the difference between the estimates obtained via the two methods increased on average as trait size decreased. This inflation of repeatability can have important consequences, potentially leading to overestimates of statistical power or parameters of interest such as heritability. Intuitively, one also might expect that soft structures would be subject to greater measurement error than sclerotized structures, and that remounting such structures would provide a more complete estimate of measurement error. In other words, as the traits in question become more difficult to measure, it becomes more important to mount specimens multiply to obtain a more realistic estimate of measurement repeatability. However, we found no evidence for significant interactions between measurement method and tissue type (soft or sclerotized), trait type (genitalic or somatic) or sex (Table 2, Figure S2 and Table S1).

Repeatability is often used to quantify the accuracy and consistency of phenotypic measurements in evolutionary and behavioral ecology. In morphometric studies, repeatabilities are often reported as a guide to the signal‐to‐noise ratio of various trait measurements. This can be useful in gauging whether a stronger effect for a particular trait, relative to other traits, might simply reflect differences among traits in measurement error (e.g., Cassidy et al., 2013). Repeatability estimates are also sometimes used to estimate the upper bounds of narrow‐ and broad‐sense heritability (Boake, 1989; Falconer and Mackay, 1996; Lynch & Walsh, 1998; Dohm, 2002). Furthermore, repeatabilities have been used to test predictions of condition‐dependent models of sexual selection. For example, Foley et al. (2012) found that for cervid antler traits, repeatability declines as environmental variation increases, supporting the idea that antlers serve as an honest signal of individual condition Our findings suggest that remounting specimens between successive measurements provide more accurate repeatability estimates in studies of morphology, especially for small structures that are difficult to measure. Furthermore, the importance of using this method, rather than simply remeasuring a single mount or image, increases as trait measurement error increases. Interestingly, while the difference between methods was small for all large structures within our sample (i.e., structures > 2.5 mm in length), the difference between methods varied considerably for the smaller structures (Figure 4). This suggests that trait size does not account fully for variation in measurement error and that other factors (e.g., trait type or degree of sclerotization) are also important. However, we did not find consistently larger differences between methods of estimating repeatability for soft traits than for sclerotized traits, genitalic traits than somatic traits, or traits in one sex. This is surprising, given that genitalic traits of insects are considered to be more difficult to measure than more rigid and sclerotized somatic structures (Ah‐King et al., 2014; Leonard & Córdoba‐Aguilar, 2010). Some, but not all genitalic structures lack well‐defined borders and landmarks, and soft tissue is easy to damage during dissection and handling (Eberhard et al., 1998). The potential for trait characteristics such as sclerotization to influence single‐mounted versus remounted repeatability warrants further investigation.

Our study adds to a growing literature illustrating that a number of factors can affect measurement error, including observer experience (Yezerinac et al., 1992), handedness of the measurer (Helm & Albrecht, 2000), the individual making the measurements (Kozlov, 2015), the interaction between instrument calibration, light, position of measured objects and experimental observer (David et al., 1999), morphometric versus distance‐based measurement methods (Takács et al., 2016), and genetic and environmental variation in the focal traits (Wilson, 2018). In studies where morphology is measured, it is important to recognize these sources of measurement error and quantify their effects. Our results suggest that remounting consistently captures more sources of measurement error and thus yields more realistic estimates of repeatability, but remounting also requires greater time and effort and increases the risk of damage to fragile samples. Thus, in deciding whether and how many times samples should be remounted, a number of factors should be taken into account. Based on our results, we suggest that traits that are difficult to measure (such as very small structures) should be remounted at least once to obtain robust estimates of measurement repeatability. For such traits, even more robust estimates of repeatability might be obtained if samples are remounted and remeasured by multiple observers. However, for traits that are relatively easy to measure (such as larger structures), remounting is less important and repeatability could be estimated from repeated measurements of a single mount to save time and effort.

In conclusion, our findings suggest that remeasuring from the same mount can yield strongly inflated repeatability estimates in morphometric studies, especially for traits subject to large measurement error. Remounting samples between measurements is likely to provide more meaningful estimates of repeatability. More broadly, the methods used to estimate repeatability should capture as many important sources of measurement error or variability as possible.

## CONFLICT OF INTEREST

None declared.

## AUTHOR CONTRIBUTION

**Zac Wylde:** Data curation (lead); Formal analysis (equal); Methodology (lead); Writing‐original draft (lead); Writing‐review & editing (equal). **Russell Bonduriansky:** Conceptualization (lead); Formal analysis (supporting); Funding acquisition (lead); Methodology (supporting); Project administration (supporting); Resources (lead); Supervision (lead); Writing‐original draft (equal).

## Supporting information

Fig S1

Fig S2

Fig S3

Appendix S1

## Data Availability

Archived data are available in the Dryad repository (https://doi.org/10.5061/dryad.hqbzkh1ds). Additionally a website presenting all of the R code used to analyze the data along with the raw data files can be found at https://github.com/wyldescience/repeatability.
