# Supplementary figures and images for: A comparison of two methods for estimating measurement repeatability in morphometric studies
Source: Ecol Evol. 2021 Jan 6;11(2):763–70. doi: 10.1002/ece3.7032 (PMC7820162; doi:10.1002/ece3.7032)

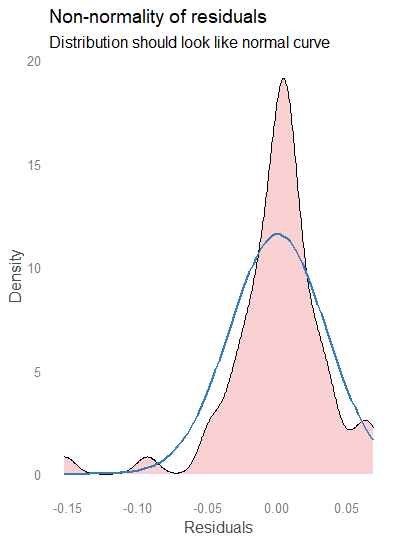

Supplement: Supplementary file 1 — Fig S1 [file ECE3-11-763-s001.png]

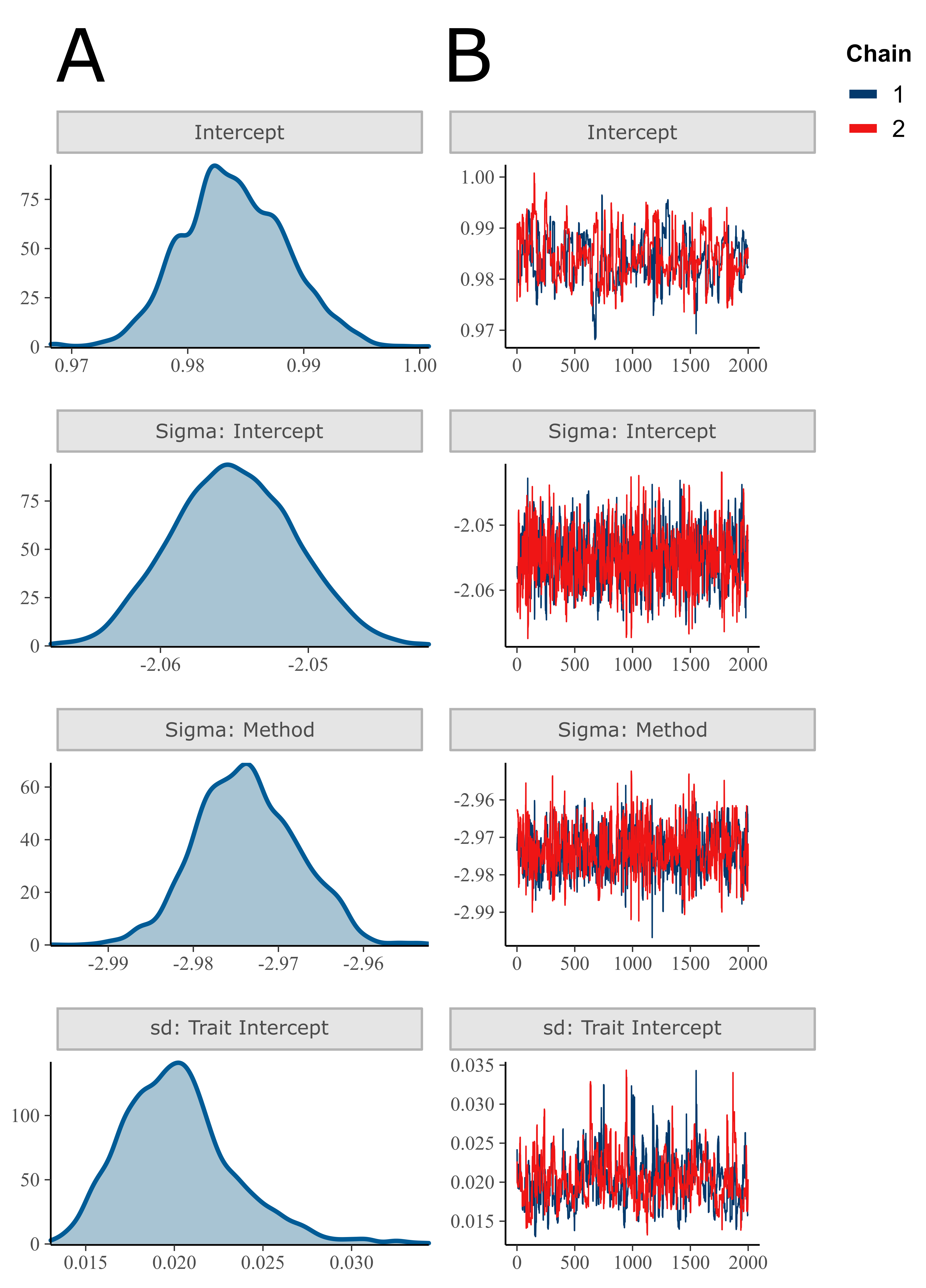

Supplement: Supplementary file 3 — Fig S3 [file ECE3-11-763-s003.png]
