# Supplementary material for: A comparison of two methods for estimating measurement repeatability in morphometric studies
Source: Ecol Evol. 2021 Jan 6;11(2):763–70. doi: 10.1002/ece3.7032 (PMC7820162; doi:10.1002/ece3.7032)

**A comparison of two methods for estimating measurement repeatability in morphometric studies**

Supplementary information

**Zachariah Wylde and Russell Bonduriansky**

**
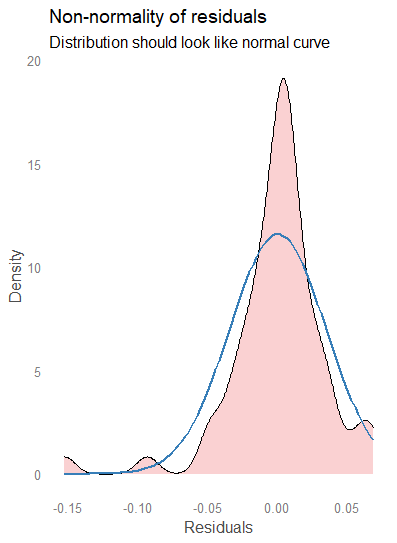
**

**Figure S1.** Distribution of residuals from the linear mixed effects model of mean repeatability estimates (see Table 2).

| Term | Df | Df.res | Sum Sq | Sum Sq.res | F value | Pr(>F) |
| --- | --- | --- | --- | --- | --- | --- |
| Method | 1 | 60 | 12124.016 | 7731.484 | 94.088 | **<0.001** |
| Sex | 1 | 60 | 128.239 | 19727.261 | 0.390 | 0.535 |
| Tissue type | 1 | 60 | 3.321 | 19852.179 | 0.010 | 0.921 |
| Trait type | 1 | 60 | 449.057 | 19406.443 | 1.388 | 0.243 |
| Method × Sex | 1 | 58 | 840.506 | 18848.077 | 2.586 | 0.113 |
| Method × Tissue type | 1 | 58 | 368.441 | 19289.220 | 1.108 | 0.297 |
| Method × Trait type | 1 | 58 | 411.615 | 19199.795 | 1.243 | 0.269 |

**Table S1.** Non-parametric tests using Aligned Rank Test non-parametric ANOVA.


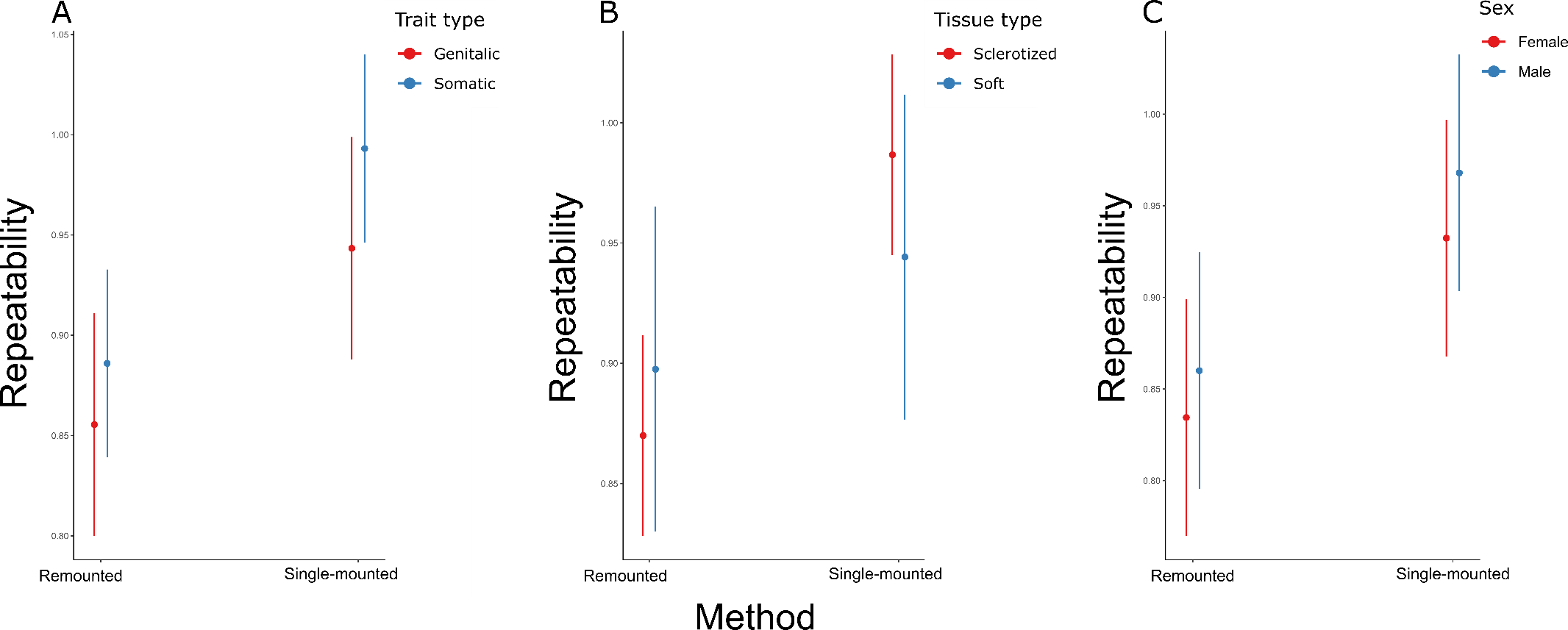


**Figure** **S2**. Estimated marginal means (predicted values) from interactions with measurement method. Panel A illustrates the interaction for trait type. Panel B illustrates interaction between tissue type. Panel C illustrates interaction between sex. All vertical bars represent 95% confidence intervals.

**Figure** **S3**. Intercepts and trace plots of Bayesian models of repeatability variance. Panel A shows the posterior distributions of each fized effect. Panel B shows the mixing of the Markov chains (MCMC). All MCMC chains converged sufficently ($\hat{R}$ = 1).


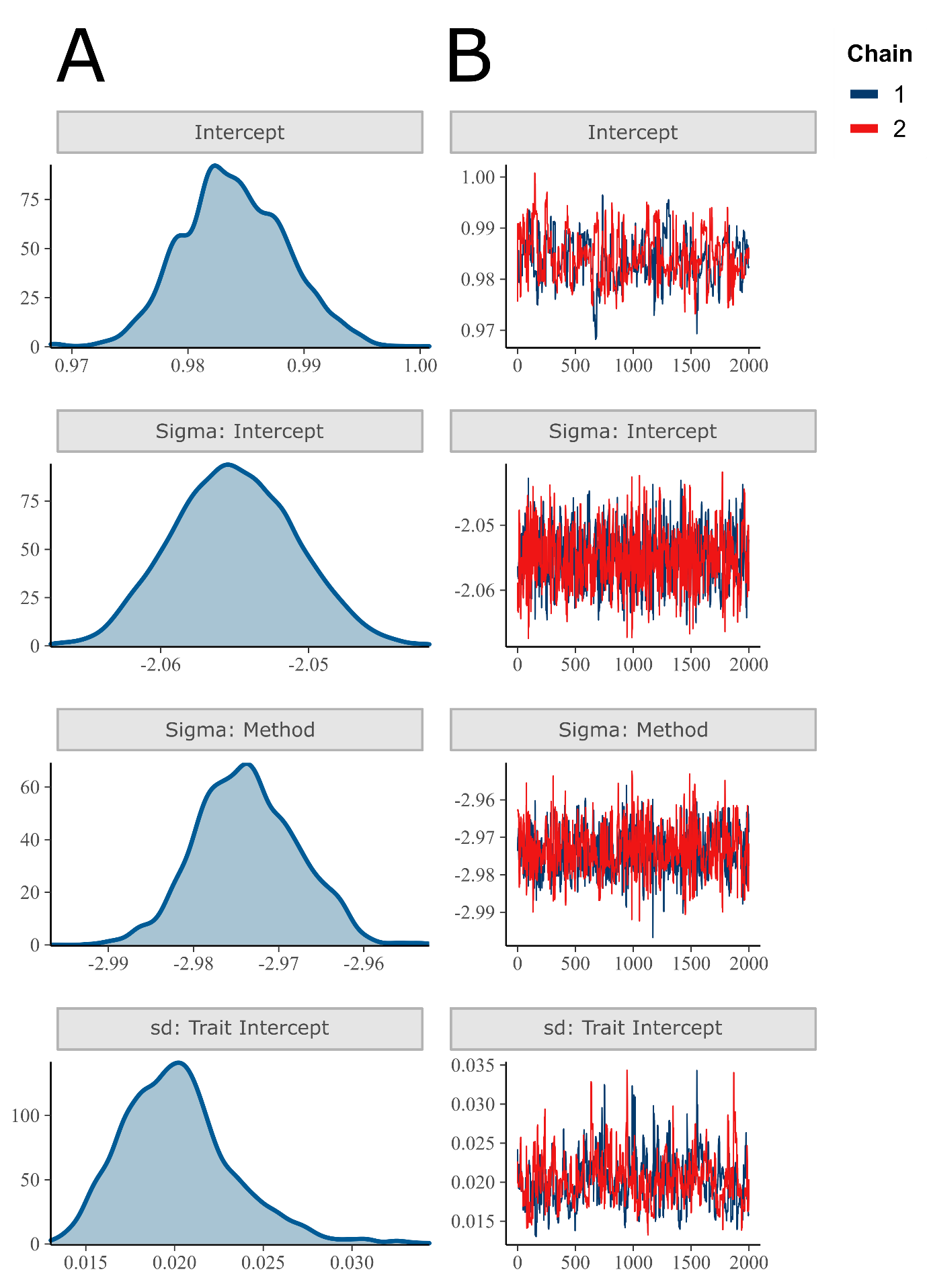


**Table S1**. Fixed parameters from Bayesian model of variance.

|  | Estimate | Est.Error | l-95% CI | u-95% CI | Rhat |
| --- | --- | --- | --- | --- | --- |
| Intercept | 0.984 | 0.004 | 0.976 | 0.993 | 1.018 |
| Sigma: Intercept | -2.055 | 0.004 | -2.063 | -2.047 | 1.002 |
| Sigma: Method | -2.974 | 0.006 | -2.985 | -2.962 | 1.003 |

**Figure** **S4**. Bayesian model of variance in repeatability as a function of method.


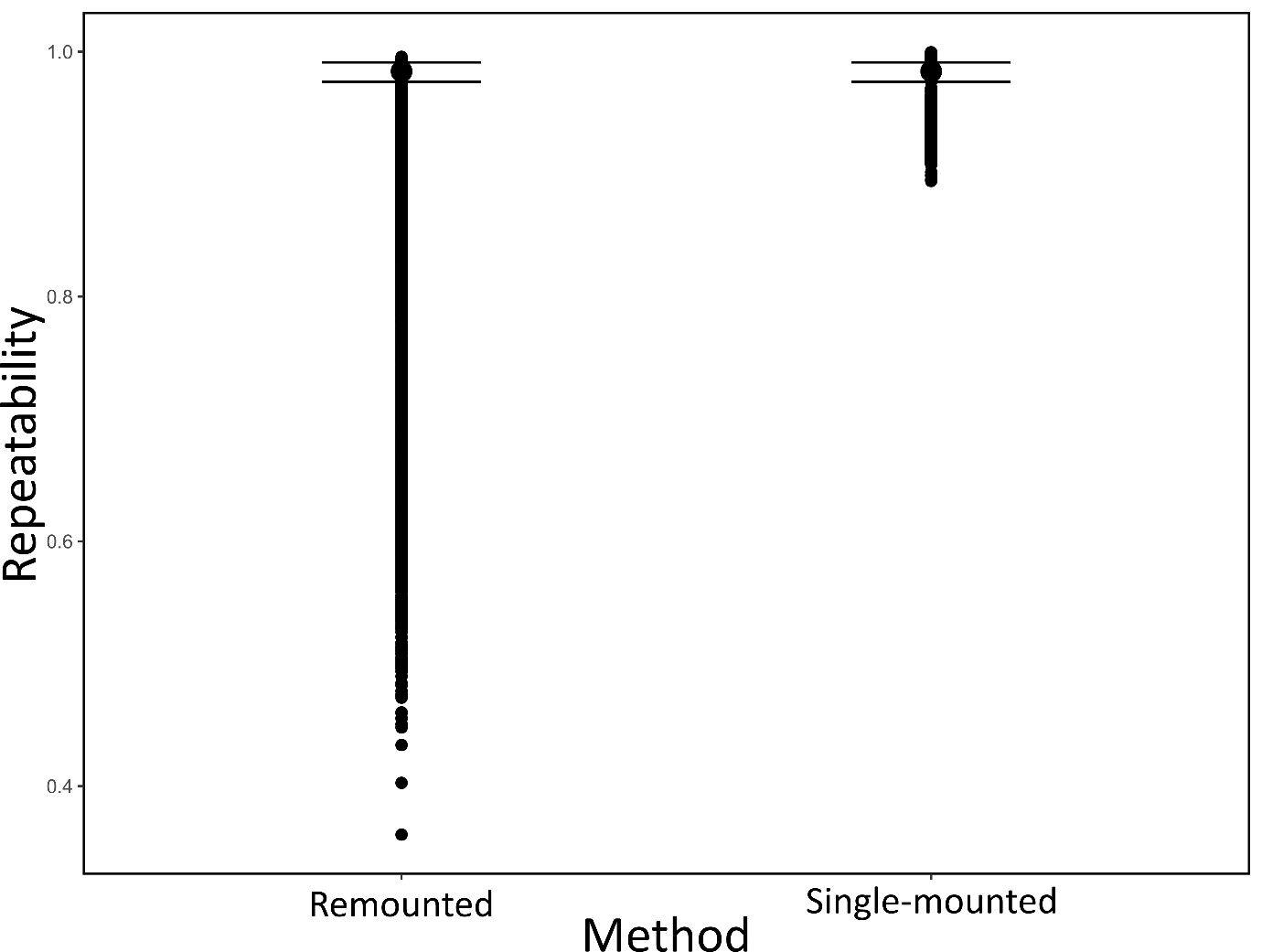

Supplement: Supplementary file 4 — Appendix S1 [file ECE3-11-763-s004.docx]
